# Differences in Weight Status and Autonomous Motivation towards Sports among Children with Various Profiles of Motor Competence and Organized Sports Participation

**DOI:** 10.3390/children8020156

**Published:** 2021-02-18

**Authors:** Eline Coppens, An De Meester, Frederik J. A. Deconinck, Kristine De Martelaer, Leen Haerens, Farid Bardid, Matthieu Lenoir, Eva D’Hondt

**Affiliations:** 1 Department of Movement and Sports Sciences, Ghent University, 9000 Ghent, Belgium; frederik.deconinck@ugent.be (F.J.A.D.); Leen.haerens@ugent.be (L.H.); farid.bardid@strath.ac.uk (F.B.); matthieu.lenoir@ugent.be (M.L.); 2 Department of Movement and Sport Sciences, Vrije Universiteit Brussel, 1050 Brussels, Belgium; kdmartel@vub.be (K.D.M.); Eva.DHondt@vub.be (E.D.); 3 Department of Physical Education, University of South Carolina, Columbia, SC 29208, USA; DEMEESTE@mailbox.sc.edu; 4 School of Education, University of Strathclyde, Glasgow G4 0LT, UK

**Keywords:** youth, BMI, cluster analyses, motor development, actual motor competence, perceived motor competence, aligned assessment tools, community sports, person-centered

## Abstract

This study aimed (1) to identify profiles in children based on actual motor competence (AMC), perceived motor competence (PMC), and organized sports participation (OSP), and (2) to examine differences among these profiles in weight status as well as autonomous motivation towards sports. Children’s (*N* = 206; 112 boys; M_age_ = 10.83 ± 0.92 years) AMC, PMC, OSP, weight status, and autonomous motivation towards sports were measured using validated assessment tools. Cluster analyses identified three profiles with completely convergent levels of AMC, PMC, and OSP and three profiles with partially convergent levels. Children in the convergent profiles with average to high levels of AMC, PMC, and OSP had the most optimal profile, as they combined a healthier weight status with elevated levels of autonomous motivation, while the opposite was true for children with low levels on all three cluster-variables. Partially convergent profiles showed that AMC and PMC appear crucial for weight status, as profiles with relatively low levels of AMC and PMC had the highest weight status, independent of their OSP levels. Overall, the findings highlight the importance of promoting AMC, PMC, and OSP simultaneously to help children in achieving a healthy weight status and being autonomously motivated towards OSP.

## 1. Introduction

**Actual motor competence** (AMC), which can be defined as the degree of proficient performance in various motor skills as well as its underlying mechanisms such as motor control and coordination [1], is associated with a range of health-related outcomes including a healthy weight status [2,3,4]. AMC is also considered important in developing an active lifestyle [5,6] since previous research has established a positive relationship between AMC and physical activity (PA) [7,8]. According to the conceptual model of Stodden and colleagues [6], a mediator in this reciprocal AMC–PA relationship is **perceived motor competence** (PMC), which refers to the self-perception of one’s AMC [9]. Both AMC and PMC are considered to be consistent predictors of PA levels more generally [5,10], and (organized) sports participation more specifically [11,12]. Likewise, PMC is found to be an important intrapersonal protective factor against dropout from **organized sports participation** (OSP) [13], while persistent OSP during childhood and adolescence is found to be a significant predictor of adult PA [14].

The positive relationship between AMC and PMC is extensively examined in the literature [15]. However, the systematic review and meta-analysis of De Meester and colleagues [15], which included data from 69 studies, showed that the strength of the relationship between AMC and PMC in children and adolescents was only low to moderate. In addition, most of the studies included in the meta-analysis only used a variable-centered approach. In this approach, the association between AMC and PMC is judged based upon a correlation between the two constructs at group level (i.e., the study sample and/or specific subsamples). In doing so, a variable-centered approach does not provide insight into how different AMC and PMC levels may be combined at the individual level [16]. Accordingly, a person-centered approach is needed to examine whether children with similar AMC levels may differ in the degree to which they perceive themselves as motor competent. Indeed, previous studies that used a person-centered approach [16,17,18,19] revealed different profiles of children, with some of them combining convergent levels of AMC and PMC (i.e., low(er) levels of AMC and low(er) levels of PMC or high(er) levels of AMC and high(er) levels of PMC), and others combining divergent levels of AMC and PMC (i.e., low(er) levels of AMC and high(er) levels of PMC, or vice versa). Yet, a limitation of prior person-centered studies is that the attributes assessed with the AMC test batteries and the PMC questionnaires are often not the same; hence, the measures of AMC and PMC are not aligned. This leaves the question whether the discrepancy in these measures may constitute one of the reasons for finding the divergent profiles.

While previous studies have shown that PA levels generally decrease in adolescence [20,21], OSP appears to be more stable over time [14,22]. Moreover, OSP in early childhood significantly increases the likelihood of continuation of OSP throughout (middle and late) childhood [23], which may promote lifelong positive pathways of health-promoting behaviors. Given the fact that OSP during childhood and adolescence has a positive effect on PA levels [11,24], this could be the way to counteract the typical decrease in PA in adolescence [20,21,24]. However, previous studies have shown that not all children have access to organized sports [25,26]. It is thus plausible that there is a group of children who do not participate in organized sports, for instance because of lower economic resources [27]. Yet, some of those children might have high levels of AMC or PMC despite a lack of OSP. In contrast, other children might be supported by their environment to participate in organized sports without necessarily having high levels of AMC or PMC. Including OSP as an additional cluster variable (next to AMC and PMC as fixed variables in earlier research) can thus provide a deeper understanding of previously identified AMC and PMC profiles. Hence, the first aim of the present study was to identify profiles in children based on AMC, PMC, and OSP while using aligned motor competence assessment tools. To this end, we examined how the three cluster variables interact, and whether convergent and divergent profiles may be identified (i.e., aim 1).

The strength of the relationship between AMC, PMC, and OSP may also relate to multiple intrapersonal characteristics, one of them being **weight status**, since all three cluster variables are negatively correlated with a higher body mass index (BMI). Estevan and colleagues [18] found that children with relatively high levels of physical capacity (i.e., AMC and physical fitness) and PMC were more likely to be normal-weight compared to those with relatively low levels of physical capacity and PMC. This is in line with the assumption of a positive spiral of engagement in the conceptual model of Stodden and colleagues [6], as well as previous research [3]. When it comes to the relationship between OSP and weight status, the evidence is rather inconclusive. While several studies established that OSP did not affect children’s weight status [11,28,29], another study suggested that OSP reduced the risk of childhood obesity regardless of the type of activity performed [30]. A person-centered approach with AMC–PMC–OSP-based profiles might unravel this ambiguity and provide new insights. Specifically, the latter approach allows one to investigate whether a higher level of one (or two) of these three cluster variables is already helpful in achieving a healthier weight status, regardless of the level of the third variable. Another intrapersonal characteristic of interest is **autonomous motivation—**the most optimal form of motivation—which involves the regulation of behavior with the experiences of volition, psychological freedom, and reflective self-endorsement [31]. Autonomous motivation for PA is associated with a variety of physical and mental health outcomes (i.e., effective performance, psychological well-being, healthy development) [32]. Children who are autonomously motivated participate in PA and sports because they enjoy doing so or because they understand and endorse the personal relevance of participation (e.g., the health benefits [32]). Moreover, AMC and PMC both are underlying mechanisms of autonomous motivation towards PA, including sports [16,33]. Children with relatively low levels of PMC were less autonomously motivated towards sports than their peers with higher levels of PMC, irrespective of their AMC level [16]. In addition, De Meester and colleagues [33] found that adolescents with relatively high levels of AMC and PMC were more autonomously motivated towards physical education than those with relatively high levels of AMC but low levels of PMC and those with relatively low levels of both. However, children with relatively low levels of AMC and high levels of PMC showed similar levels of autonomous motivation as their peers with high levels of both constructs. These studies thus suggest that higher levels of PMC could compensate for lower levels of AMC in terms of autonomous motivation towards sports [16] or physical education [33]. In addition, the study of De Meester and colleagues [33] revealed that students with relatively low levels of AMC and PMC were less autonomously motivated and were also less involved in OSP than their peers with higher levels of AMC and/or PMC. Taking into account these findings, identifying profiles based on AMC-PMC-OSP might reveal a group of children showing low levels of AMC, but high levels of PMC and OSP, who are indeed autonomously motivated towards sports. It seems, then, that understanding whether and how various profiles based on AMC–PMC–OSP differently relate to autonomous motivation for sports can help us in further promoting engagement in PA and sports in order to stimulate a healthy development. Therefore, the second aim of the current study was to compare the AMC–PMC–OSP profiles in terms of weight status and autonomous motivation towards OSP (i.e., aim 2). Based on studies using the person-centered approach [16,18,19], it was hypothesized that children with relatively high levels of AMC, PMC, and OSP would have a healthier weight status [18] and a higher autonomous motivation towards OSP [16] when compared to children with relatively low levels of AMC, PMC, and OSP. It was also hypothesized that children with relatively high levels of PMC and OSP, but low levels of AMC, would be more autonomously motivated towards sports compared to children with the opposite profile (i.e., low levels of PMC and OSP, but high levels of AMC) [16,33,34].

## 2. Materials and Methods

### 2.1. Participants and Procedure

A sample of 206 children (112 boys) with a mean age of 10.83 years (SD = 0.92, range 9.07–12.95 years) agreed to participate in the current cross-sectional study. Data collection took place during weekends and school breaks (between August 2018 and February 2019) and took approximately two hours per participant. Each session consisted of completing anthropometric measurements, an AMC test battery, and a questionnaire assessing PMC as well as weekly participation time in organized sports (see below for details), and autonomous motivation towards organized sports. Test administration was conducted by experienced examiners conducting the assessments using standardized instructions in accordance with the AMC test manual [35]. Participants wore light sports clothing and were barefooted to ensure uniformity of test conditions. When completing the questionnaire, participants had the opportunity to ask for clarification whenever necessary. In addition, a visual demonstration was given to illustrate the PMC items to the children. Written informed consent to participate in the current study was provided for each child by their parent(s) or legal guardian. The study protocol was approved by the Ethics Committee of Ghent University Hospital.

### 2.2. Measurements

#### 2.2.1. Actual Motor Competence

The Körperkoordinationstest für Kinder (KTK) [35,36] was used to evaluate children’s AMC level. It is a standardized, normative, product-oriented test battery to assess AMC in terms of gross motor coordination in 5- to 15-year-old children with typical or atypical motor development. The KTK is a highly reliable instrument with excellent test–retest reliability for the total raw score (*r* = 0.97), and very good inter- and intra-rater reliability for the subtest raw scores (*r* values > 0.85 and ranging between 0.80 and 0.96, respectively) [35,36]. In addition, it also a valid instrument, showing moderately strong correlations with other standardized AMC assessment tools [23,37,38,39]. The KTK test battery includes four subtests (i.e., walking backwards, moving sideways, jumping sideways, and hopping for height), and takes approximately 20 min per participant to complete. The raw scores of each subtest are converted into standardized scores adjusted for age (all subtests) and sex (walking backwards, jumping sideways, and hopping for height). These standardized scores are then summed to compute an overall motor quotient (MQ), using the KTK manual’s normative tables based on the performance of the reference sample [35].

#### 2.2.2. Perceived Motor Competence

An adapted version of the Physical Self-Confidence Scale (PSCS) [40] was used to assess children’s PMC. The original PSCS contains 15 items for which participants rate their perceived self-confidence in performing specific motor skills on a 10-point Likert scale, ranging from “being not confident at all” (=1) to “being very confident” (=10). These items are aligned with the locomotor and object control skills assessed in the Test of Gross Motor Development-2 (TGMD-2) [41,42] and the balance skills assessed in the Victorian Fundamental Movement Skills Test [43]. The PSCS’s test–retest reliability is considered excellent with an overall intra class correlation of 0.92 [40]. Content validity and concurrent validity are also good, with the scale achieving a correlation coefficient of 0.72 with the Physical Self-Perception Profile [40,44]. Following expert advice, and since the aim was to measure perceived competence rather than self-confidence, the question stem of the items was altered from “how confident are you at performing” to “how well can you perform” [45]. For the purpose of the present study, four items were added to this PSCS questionnaire, in alignment with the four KTK subtests (i.e., “*How well can you perform walking backwards on a balance beam*?”, “*How well can you perform moving sideways as fast as possible with the aid of two wooden boards?*”, “*How well can you perform jumping sideways over a slat as fast as possible?*”, “*How well can you perform hopping for height on one leg over an increasing number of foam squares?*”). For the current study, the participant’s PMC subscore (ranging from 1 to 10) was determined by calculating the average score of the four items that were aligned with the four KTK subtests. To estimate the reliability of these four additional items, a test–retest procedure was performed. To this end, 64 children between 9 and 11 years of age completed these questions twice in similar conditions, with a 19-day interval, showing a moderate degree of reliability with an ICC of 0.78 and a 95% confidence interval ranging from 0.66 to 0.86 (F (60,60) = 8.181, *p* < 0.001). Convergent validity was established through a significant positive relationship between the PMC subscore based on the original 15 PSCS items and the PMC subscore based on the four KTK items, using Pearson’s correlation (*r* = 0.63, *p* < 0.001).

#### 2.2.3. Organized Sports Participation

General information about children’s participation in organized sports was obtained using sections of the Flemish Physical Activity Questionnaire (FPAQ) [46], which has shown to be a reliable instrument (test–retest reliability coefficients ranging from 0.69 to 0.93) to assess different dimensions of habitual PA.

#### 2.2.4. Weight Status

Participants’ body height was measured using a portable stadiometer with an accuracy of 0.1 cm (Harpenden, Holtain Ltd., Crymych, UK), and their body weight was determined by means of a digital scale with an accuracy of 0.1 kg (Seca, Model 770, Hamburg, Germany). These measures were combined to compute children’s BMI (kg/m²). Next, reference population-based BMI z-scores (zBMI) were computed based on the Flemish growth curves to obtain a relative measure of adiposity adjusted for sex and age [47], which was used as an estimate of children’s weight status.

#### 2.2.5. Autonomous Motivation for Sports

Children’s autonomous motivation towards sports was assessed using an adapted (i.e., age-appropriate) version of the Behavioral Regulation in Exercise Questionnaire (BREQ), containing 12 items, which was validated in previous research in a sample of children with a similar age range as our sample [48]. For the purpose of the present study, only the six items regarding autonomous motivation were measured. Each of these six items starts with the stem “*I participate in organized sports because*…”. The items relate to identified regulation (e.g., “*I participate in organized sports because it is important for me to participate in organized sports*”, 3 items), and intrinsic regulation (e.g., “*I participate in organized sports because participating in organized sports is fun*”, 3 items). Participants responded to each of the items via a 5-point Likert scale, ranging from “not at all true for me” (= 1) to “very true for me” (= 5). Each participant’s autonomous motivation subscore (also ranging from 1 to 5) was determined by calculating the average score of both the identified regulation (3 items) and intrinsic regulation (3 items) subscales of the adapted BREQ.

### 2.3. Statistical Analysis

All statistical analyses were performed using IBM SPSS statistics version 26 (IBM Corporation. Armonk. NY. USA) with *p*-values below 0.05 being considered as statistically significant.

As a preliminary step, the relationship between all study variables (i.e., both cluster and outcome variables) was examined by means of Spearman’s rank correlation coefficients. Correlation coefficients were interpreted as negligible: <0.30, low: 0.30–0.50, moderate: 0.50–0.70, high: 0.70–0.90, or very high: 0.90–1.00 [49].

Cluster analyses were conducted based on the participating children’s **AMC, PMC, and OSP** scores to examine whether different profiles could be identified based on these three **cluster variables** (i.e., aim 1). After standardizing the scores of AMC, PMC, and OSP (i.e., conversion into sample-based z-scores), six univariate outliers were removed (i.e., with an absolute z-score of more than three). Using the Mahalanobis distance measure, one additional multivariate outlier had to be removed, resulting in a final sample of 199 children. Next, a two-step procedure of hierarchical and non-hierarchical clustering methods was applied on AMC, PMC, and OSP z-scores [50], and Ward’s hierarchical clustering method was conducted to combine clusters based on similarity of squared Euclidean distance [49]. This analysis resulted in a three, four-, five-, and six-cluster solution. If the explained variance within a cluster solution was less than 50% for AMC, PMC, and/or OSP, the cluster solution was eliminated from the following step [51]. As a result, the three- and four-cluster solutions were eliminated (based on an explained variance for OSP of 48.8% and 30.2%, respectively). Cluster centers were then used as non-random initial cluster centers in an iterative, non-hierarchical *k*-means clustering procedure [52]. After that, a double-split cross-validation procedure was conducted to explore the stability of the cluster solutions by randomly splitting the dataset into halves and applying the two-step procedure of Ward and k-means in each subsample [53]. The children in the first half were again clustered based on their Euclidean distances to the cluster center of the other half. The new and original clusters were compared for agreement by means of Cohen’s kappa. A Cohen’s kappa of >0.60, indicating good agreement of the averaged two resulting kappa’s, was considered as acceptable [52]. The six-cluster solution had a higher kappa value (0.742) than the five-cluster solution (0.404). Therefore, only the six-cluster solution was used for further interpretation, which explained 71.4%, 61%, and 63.6% of the variance in AMC, PMC, and OSP, respectively.

A Chi-square test was subsequently conducted to explore whether the sex distribution in the clusters matched the sex distribution in the total sample, and independent-samples t-tests were conducted to examine whether boys and girls significantly differed from each other in terms of the **outcome variables** (i.e., **weight status** and **autonomous motivation towards sports**). Furthermore, a one-way ANOVA was conducted to analyze potential age-related differences in the cluster and outcome variables. 

To investigate differences in **weight status** (i.e., zBMI) and **autonomous motivation towards sports** among the six clusters (i.e., aim 2), a one-way MANOVA was conducted. Bonferroni adjusted post hoc analyses were used to detect significant subgroup (i.e., Profile) differences.

## 3. Results

### 3.1. Descriptives and Correlations

Table 1 presents the means and standard deviations of both the cluster and outcome variables, as well as the correlation coefficients among these variables. 

### 3.2. Identifying Profiles

**Figure 1:**
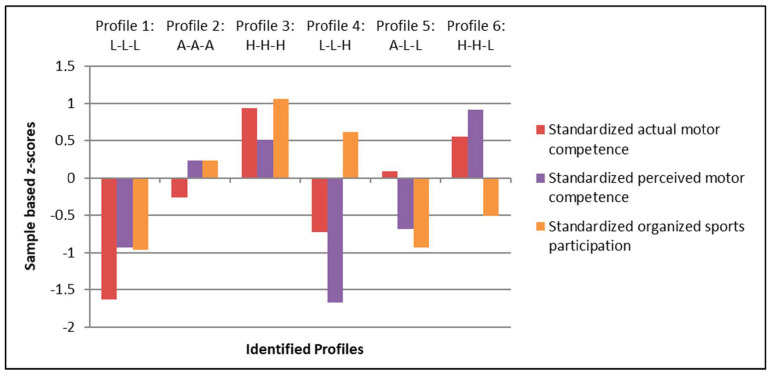
Identification of six profiles based on sample-based z-scores for actual motor competence (AMC), perceived motor competence (PMC), and organized sports participation (OSP) (L: low, A: average, H: high).

Cluster analyses revealed six different profiles, which are shown in Figure 1. These identified profiles were labelled based on relative (i.e., compared to the study sample) levels of AMC (high–average–low), PMC (high–average–low), and OSP (high–average–low), respectively. A cluster variable (i.e., AMC, PMC, OSP) was labelled as high when the z-score was above +0.50, as average when the z-score was equal to or between −0.50 and +0.50, and as low when the z-score was below −0.50.

Six profiles were identified, of which three profiles had completely convergent levels of AMC, PMC, and OSP (i.e., “low-low-low”, “average-average-average”, “high-high-high”), representing 52.2% (*n* = 104) of the total sample. Furthermore, three profiles with partially convergent levels of AMC, PMC, and OSP were found (i.e., “low-low-high”, “average-low-low”, “high-high-low”), representing 47.8% (*n* = 95) of the study sample.

Table 2 represents the means and standard deviations of the three cluster variables (i.e., AMC, PMC, and OSP) as well as both outcome variables (i.e., weight status (zBMI) and autonomous motivation) for each of the six identified profiles. Profile 1 (L–L–L; *n* = 20; 40.0% boys) consisted of children who had relatively low levels of AMC, PMC, and OSP when compared to children belonging to the other profiles. Profile 2 (A–A–A; *n* = 43; 69.7% boys) was characterized by children who displayed relatively average levels of AMC, PMC, and OSP when compared to children belonging to the other profiles. Children in Profile 3 (H–H–H; *n* = 41; 58.5% boys) showed relatively high levels of AMC, PMC, and OSP when compared to children belonging to the other profiles. A minority of children (i.e., Profile 4; L–L–H; *n* = 17; 58.8% boys) was characterized by children who had relatively low levels of AMC and PMC but relatively high levels of OSP when compared to children belonging to the other profiles. Children in Profile 5 (A–L–L; *n* = 32; 56.2% boys) displayed a relatively average level of AMC and relatively low levels of PMC and OSP when compared to children belonging to the other profiles. Finally, Profile 6 (H–H–L; *n* = 46; 41.3% boys) consisted of children who showed relatively high levels of AMC and PMC but a low level of OSP when compared to children belonging to the other profiles. The three groups of children with relatively average or high levels of both AMC and PMC (i.e., Profile 2, Profile 3, and Profile 6) were the three biggest groups, together accounting for 65.3% of the total sample, while the group of children with relatively low levels of AMC and PMC and high levels of OSP was the smallest group, accounting for only 8.5% of the total sample.

### 3.3. Differences in Weight Status and Autonomous Motivation among Profiles

Preliminary analyses were conducted to check for sex and age-related differences among clusters and outcome variables. A Chi-square test showed a similar sex distribution among each of the six identified profiles (χ^2^ (5) = 9.408, *p* = 0.094, Φ_Cramer_ = 0.217). Regarding age, a one-way ANOVA indicated a significant age-effect (F = 2.677, *p* = 0.023, η^2^ = 0.065). However, Bonferroni post hoc tests only revealed a significant, almost negligible, difference in age (*p* = 0.049) between the younger children in Profile 5 (i.e., “average–low–low”, mean age = 10.51 years) and the slightly older children in Profile 3 (i.e., “high–high–high”, mean age = 11.15 years). With respect to the outcome variables, independent-sample t-tests revealed no significant differences between boys and girls in weight status (t (197) = –0.138, *p* = 0.890, *r* = 0.098) and autonomous motivation towards sports (t (197) = –0.203, *p* = 0.840, *r* = 0.014) in the total sample. In addition, weight status (F = 2298; *p* = 0.079; partial η^2^ = 0.034) and autonomous motivation towards sports (F = 1.357; *p* = 0.257, partial η^2^ = 0.020) did not differ between age groups (i.e., 9–9.99, 10–10.99, 11–11.99, 12–12.99 years). Therefore, both sex and age were not taken into consideration in the subsequent analyses.

The one-way MANOVA showed significant differences among the identified profiles for weight status (F = 6.744, *p* < 0.001) and autonomous motivation (F = 9.063, *p* < 0.001). With respect to **weight status** (i.e., zBMI), children in the “high–high–high” profile (i.e., Profile 3), children in the “average–low–low” profile (i.e., Profile 5), and children in the “high–high–low” profile (i.e., Profile 6) demonstrated a significantly lower zBMI than children in the “low–low–low” profile (i.e., Profile 1) and children in the “low–low–high” profile (i.e., Profile 4). No other differences in weight status between the previously mentioned profiles were established, and no significant zBMI differences were found between children in the “average–average–average” profile (i.e., Profile 2), and any of the other profiles. **Autonomous motivation** towards organized sports was significantly higher in the “high–high–high” profile (i.e., Profile 3) when compared to the other profiles, with the exception of the children in the “average–average–average” profile (i.e., Profile 2). Children in the “low–low–low” profile (i.e., Profile 1), the “low–low–high” profile (i.e., Profile 4), and the “average–low–low” profile (i.e., Profile 5) scored significantly lower on autonomous motivation when compared to children in the “average–average–average” profile (i.e., Profile 2) and children in the “high–high–high” profile (i.e., Profile 3).

## 4. Discussion

The present study used a person-centered approach to identify various profiles based on children’s AMC, PMC, and OSP (i.e., aim 1). In addition, it was examined how children in the various AMC–PMC–OSP-based profiles may differ from each other in terms of weight status (i.e., zBMI) and autonomous motivation towards sports (i.e., aim 2). We addressed these aims in a study sample with AMC levels that were slightly above average (i.e., MQ of 107 versus the reference value of 100) and PMC levels that were comparable to the PMC levels in an older sample [40]. When it comes to OSP, our study sample showed higher levels when compared to the reported OSP levels in a previous study in the same age category [54]. In terms of outcome variables (i.e., weight status and autonomous motivation), our study sample was comparable with other studies conducted in children within the same age range [16,54].

### 4.1. AIM 1. Identifying Profiles Based on Children’s AMC, PMC, and OSP

The cluster analyses revealed three profiles with completely convergent levels of AMC, PMC, and OSP (i.e., “low–low–low”, “average–average–average”, “high–high–high”), and three profiles with partially convergent levels thereof (“low–low–high”, “average–low–low”, “high–high–low”). When only considering AMC and PMC in the current sample, 5 out of 6 profiles (83.9% children of the total sample) had convergent levels of AMC and PMC, and only one profile (i.e., Profile 5) had divergent levels of AMC and PMC. In that respect, our findings differ from prior studies which revealed three [19] or four [16,33] motor competence based profiles: one profile with relatively low levels of both AMC and PMC (i.e., “low–low”), one profile with relatively high scores of both constructs (i.e., “high–high”), one profile combining relatively low levels of AMC with relatively high levels of PMC (i.e., “low–high”), and one profile combining relatively high levels of AMC with relatively low levels of PMC (i.e., “high–low”). Interestingly, we did not clearly identify a “low–high” or “high–low” profile. However, Profile 2 (“average–average–average”) and Profile 5 (“average–low–low”) would have been considered as divergent profiles if we would have used the cut-off points of the previous studies (i.e., low (i.e., below average) or high (i.e., above average)) based on AMC and PMC [16,19,33]. Another explanation for the majority of children having convergent AMC and PMC levels in the current study may be that we used an aligned (i.e., with the AMC test battery) product-oriented measure of PMC. The four KTK-related items in the PMC questionnaire were directly aligned with the test items of the AMC assessment tool (i.e., KTK), which may explain why we found a relatively stronger relationship between AMC and PMC in this study when compared to previous studies focusing on middle childhood [15]. Not only the alignment, but also the nature of the assessment tools, which were both product-oriented, may have resulted in a stronger relationship between AMC and PMC. It is possible that children have less difficulties with feeding product-oriented information back to themselves (e.g., *“How many steps did I walk backwards on the balance beam?”*) when compared to process-oriented information (e.g., *“Did I extend my arm while reaching for the ball as it arrives?”*) [55]. Children may also have used the product-oriented feedback to provide additional relevant information about their self-perceived level of skill in comparison to process-oriented feedback [55], resulting in a stronger correlation between the aligned AMC and PMC in the current study when compared to earlier research also using a person-centered approach [16,18,19,56].

The addition of OSP as a third cluster variable yielded some additional and interesting insights that warrant further discussion. First, there appeared to be a small group of children (i.e., Profile 4) with relatively low levels of AMC and PMC but relatively high levels of OSP (i.e., “low–low–high”). This may indicate that having low levels of AMC and PMC does not necessarily prevent children from taking part in organized sports, which is in contrast with previous literature [5,10,33]. On the other hand, the expected positive effect in the other direction, with more OSP being associated with higher levels of AMC [57] and PMC [58] also seems to be absent in Profile 4. One explanation may be that children in this “low–low–high” profile are supported by their environment to participate in organized sports without necessarily having high levels of AMC or PMC. There was also a group of children, constituting of 23.1% of the sample (i.e., Profile 6), who had relatively high AMC and PMC levels but did not frequently participate in OSP (i.e., “high–high–low”). A possible explanation for the (unexpected) low levels of OSP, which is in contrast to their high levels of AMC and PMC, may be that these children are physically active (e.g., active free play, active transport) and even participate in unorganized sports (i.e., running, swimming, etc.), but not necessarily in sports in an organized setting. Altogether, these findings demonstrate the added value of a person-centered approach and including OSP as an additional third cluster variable. Previous research has shown that several factors play a role in OSP (e.g., gender, socio-economic status, social support from parents and friends, and economic resources) [26,59]. Moreover, psychosocial barriers and facilitators in OSP might explain the differences in the divergent profiles. Therefore, further research exploring psychosocial factors such as socio-economic status and parental support as well as enjoyment, and the relationship of each of these factors with OSP (and by extension AMC and PMC), could be helpful to further unravel the identified profiles.

### 4.2. AIM 2. Examining Differences in Weight Status and Autonomous Motivation According to AMC–PMC–OSP Profiles

First, taken together all findings, the children with average to high levels on all three variables (i.e., Profile 2 and 3) displayed the most optimal profile, as they had both low levels of zBMI (representing a healthier weight status) as well as high levels of autonomous motivation towards sports, while the opposite was true for children with low levels of all three variables (i.e., Profile 1). These findings align with previous studies revealing that children with relatively low levels of AMC and PMC were more likely to have a higher BMI (representing a less healthy weight status) compared to those with relatively high levels of AMC and PMC [18,19], and with evidence that suggests that low levels of AMC and PMC is the least optimal combination in terms of autonomous motivation [16,33].

Some interesting patterns further emerge when considering the divergent profiles. In terms of **weight status**, the two profiles with the lowest levels of AMC and PMC (i.e., Profile 1 and 4) revealed the highest zBMI, independent of whether they had low (i.e., Profile 1) or high (i.e., Profile 4) OSP levels. Our findings thus show that being involved in OSP does not necessarily compensate for low levels of AMC and PMC in terms of a healthy weight status. In fact, our study seems to indicate that more desirable levels of zBMI are associated with average (i.e., Profile 2) or high (i.e., Profile 3 and 6) levels of both AMC and PMC, further stressing the importance of promoting both AMC and PMC for a healthy weight status. In terms of **autonomous motivation,** findings clearly point towards the importance of promoting average to high levels of AMC, PMC, and OSP simultaneously. While previous research revealed that relatively high levels of PMC can compensate for relatively low levels of AMC in children [16] as well as in adolescents [33,60], this hypothesis could not be tested in the current study as we did not identify a group of children with relatively low AMC levels and relatively high PMC levels. However, a closer look at the profiles reveals that at least some children in Profile 2 (“average–average–average”) may have low(er) levels of AMC and high(er) levels of PMC, while having an average level of OSP. Finally, it was surprising to note that the group with average levels of AMC and low levels of PMC and OSP (i.e., Profile 5), which would be considered a less optimal profile, still had relatively low levels of zBMI. Yet, the latter group displayed lower levels of autonomous motivation. Thus, they were less likely to value and enjoy OSP, hereby displaying a mixed pattern of outcomes, which is in line with the findings of Estevan et al. [60].

### 4.3. Strengths and Limitations, and Recommendations for Future Research

A major strength of the present study is the use of a person-centered approach, which enabled a deeper understanding of how AMC, PMC, and OSP are combined within 9- to 13-year-old children, and in turn relate to their weight status and autonomous motivation towards sports. As stated by Cairney et al. [61], incorporating other domains than AMC and PMC in the person-centered approach allows one to more profoundly examine the interrelationship among different health-related outcomes. Another strength is the use of aligned assessment instruments for the measurement of AMC and PMC. Using such measures that capture constructs in a similar way can help us to gain more insight into how children’s perceptions of motor competence correspond to their AMC. In future studies, combinations of aligned and product- and process-oriented measures of PMC could be incorporated to examine how profiles and findings may differ depending on the type of measurement being used. Despite the strengths of this study, it must be taken into account that this study has a cross-sectional design. Therefore, the results do not provide any causal evidence regarding significant relationships that were found among study variables. Longitudinal and experimental studies should be conducted to gain more insight in the direction of these relationships. Furthermore, differences among the six identified profiles could have been less pronounced as group sizes were not equal, ranging from 17 to 46 participants per profile, but since the study sample was sufficiently large to perform cluster analyses [53], results are to be considered legitimate. However, further research with a larger sample is recommended to support the identified cluster-profiles of the current study. Another limitation of this study is that only one specific type of PA (i.e., OSP) was included as a measurement of PA. Future research would benefit from also including measurements of other types of PA, such as participation in unorganized sports, active transport, and school-based PA. In addition, using the KTK as AMC test battery does not provide a comprehensive picture for motor competence, since it mainly measures stability and locomotion [62]. Finally, this study can set the stage for examining psychosocial and environment antecedents of children’s profiles to examine which factors (e.g., socio-economic status, parental support) explain why children belong to a certain profile.

## 5. Conclusions

The present study identified three completely convergent and three partially convergent AMC–PMC–OSP-based profiles in 9- to 13-year-old children. Results revealed that children with average to high levels on all three variables displayed the most optimal health-related profile as they had (on average) a healthier weight status as well as higher levels of autonomous motivation, while the opposite was true for children with low levels of all three variables. Our findings further showed that being involved in OSP does not necessarily compensate for low levels of AMC and PMC in terms of a healthy weight status. The results of the present study also highlight the importance of targeting and monitoring AMC and PMC in physical education and sports settings to help children in achieving a healthy weight status. These endeavors might result in a healthier weight status and higher levels of autonomous motivation towards sports, both essential aspects of a healthy and active lifestyle.

## Figures and Tables

**Table 1:** Means and standard deviations of the cluster (i.e., 1-3) and outcome (i.e., 4-5) variables (*N* = 199, 109 boys) and correlations among variables.

		Mean	SD	Min	Max	1	2	3	4
	**Cluster Variables**								
1	AMC (MQ)	107.32	14.44	60	138				
2	PMC *(scale 1–10)*	7.32	1.44	1	10	0.502 **			
3	OSP *(hours/week)*	2.52	1.24	0	6.25	0.308 **	0.137		
	**Outcome Variables**								
4	BMI z-score	−0.14	0.95	−2.29	2.52	−0.292 **	-0.197 *	0.057	
5	Autonomous motivation *(scale 1–5)*	4.31	0.73	1	5	0.302 **	0.333 **	0.405 **	−0.079

AMC: actual motor competence; MQ: motor quotient; PMC: perceived motor competence; OSP: organized sports participation; BMI: body mass index; SD: standard deviation; Min: minimum; Max: maximum ** *p* < 0.001; * *p* < 0.01.

**Table 2:** Identified profiles: Means and standard deviations of the cluster variables (AMC, PMC, OSP) and outcome variables (BMI, autonomous motivation) for the six profiles (*N* = 199).

	Profile 1 L-L-L	Profile 2: A-A-A	Profile 3 H-H-H	Profile 4 L-L-H	Profile 5 A-L-L	Profile 6 H-H-L
	*n* = 20 8 Boys	*n* = 43 30 Boys	*n* = 41 24 Boys	*n* = 17 10 Boys	*n* = 32 18 Boys	*n* = 46 19 Boys
**Cluster variables (z-scores)**						
Actual motor competence	−1.63 ± 0.56 ^a^	−0.26 ± 0.49 ^c^	0.93 ± 0.42 ^f^	−0.73 ± 0.61 ^b^	0.09 ± 0.39 ^d^	0.55 ± 0.46 ^e^
Perceived motor competence	−0.93 ± 0.61 ^b^	0.23 ± 0.42 ^c^	0.51 ± 0.66 ^c^	−1.67 ± 0.52 ^a^	−0.68 ± 0.66 ^b^	0.91 ± 0.41 ^d^
Organized sports participation	−0.96 ± 0.63 ^a^	0.23 ± 0.44 ^c^	1.06 ±0.58 ^d^	0.61 ± 0.68 ^c,d^	−0.93 ± 0.48 ^a^	−0.51 ± 0.49 ^b^
**Cluster variables (raw scores)**						
Actual motor competence	80.10 ± 0.91 ^a^	102.26 ±7.92 ^c^	121.59 ± 6.80 ^f^	94.71 ± 9.81 ^b^	107.94 ± 6.29 ^d^	115.39 ± 7.45 ^e^
Perceived motor competence	5.91 ± 0.91 ^b^	7.64 ± 0.76 ^c^	8.04 ± 0.98 ^c^	4.81 ± 0.77 ^a^	6.28 ± 0.97 ^b^	8.64 ± 0.62 ^d^
Organized sports participation	1.29 ± 0.86 ^a^	2.90 ± 0.60 ^c^	4.02 ± 0.79 ^d^	3.41 ± 0.91 ^c,d^	1.31 ± 0.65 ^a^	1.90 ± 0.66 ^b^
**Outcome variables**						
Body mass index (BMI) z-score	0.50 ± 1.25 ^b,c^	0.02 ± 0.89 ^a,b,c^	−0.39 ± 0.66 ^a^	0.57 ± 1.17 ^c^	−0.45 ± 0.79 ^a^	−0.39 ± 0.82 ^a^
Autonomous motivation for sports	3.72 ± 0.82 ^a^	4.56 ± 0.44 ^c,d,e^	4.70 ± 0.38 ^e^	4.13 ± 0.70 ^a,b^	3.99 ± 0.72 ^a,b^	4.27 ± 0.87 ^b,c^

L: low; A: average; H: high; ^a,b,c,d,e,f^: A cluster mean is significantly different (*p* < 0.05) from another mean if they have different superscripts. Differences between the six profiles were tested by Bonferroni adjusted post hoc analyses.

## Data Availability

The data presented in this study are openly available in Open Science Framework at https://osf.io/ry9sc/?view_only=3771b77bb46b490eab2c9d82c56007a9 (accessed on 18 February 2021).
